# Application of Acupoint Catgut Embedding Therapy Combined with Liuzijue Breathing Exercise in the Treatment of Patients with Stable Chronic Obstructive Pulmonary Disease

**DOI:** 10.1155/2022/4084505

**Published:** 2022-10-06

**Authors:** Yun Zheng, Jing Xia, Jisheng Zheng

**Affiliations:** Pulmonary and Critical Care Medicine, Tongde Hospital of Zhejiang Province, Hangzhou, Zhejiang 310012, China

## Abstract

**Objective:**

To evaluate the application value of acupoint catgut embedding therapy combined with Liuzijue breathing exercise in the treatment of patients with stable chronic obstructive pulmonary disease (COPD) and its impact on immune function and quality of life.

**Methods:**

A total of 100 patients with stable COPD admitted to our hospital from February 2020 to February 2021 were included and assigned to the experimental group (*n* = 50) and the control group (*n* = 50) according to the order of admission. Both groups of patients received conventional treatment. The control group was given daily inhalation of budesonide and formoterol fumarate powder for inhalation (320 ug/bottle), and the experimental group received additional acupoint catgut embedding therapy combined with Liuzijue breathing exercise. The clinical efficacy, pulmonary function indexes, activities of daily living (ADL) scores, quality of life (QOL) scores, traditional Chinese medicine (TCM) syndrome scores, the number of acute exacerbations, medical expenses, the incidence of adverse reactions, and immune indicators were compared between the two groups of patients.

**Results:**

The experimental group yielded a significantly higher effective rate of treatment than the control group (*P* < 0.05). After the treatment, the experimental group obtained a superior outcome in terms of lung function indexes, immune function indexes, ADL and QOL scores, and the TCM syndrome scores when compared with the control group (*P* < 0.05). The number of acute exacerbations in the experimental group was remarkably lower than that in the control group (*P* < 0.05). No serious adverse reactions were observed in the two groups of patients, and no significant difference in the incidence of adverse reactions was found (*P* > 0.05).

**Conclusion:**

Acupoint catgut embedding therapy combined with Liuzijue breathing exercise, with high safety, can improve the treatment effect and the quality of life of patients with stable COPD, which merits clinical promotion.

## 1. Introduction

Chronic obstructive pulmonary disease (COPD) is a common clinical chronic disease that is characterized by persistent airflow limitation. Lung inflammation can be triggered by harmful gases or dust particles, with symptoms of varying degrees of coughing, chest tightness, and dyspnea, which seriously impedes the patient's quality of life [1–3]. Currently, the morbidity of COPD for residents over 40 years in China is 8.2%, while its mortality rate ranks first in the world [4, 5]. The reason mainly lies in patients' underestimation of the significance of treatment in the stable period, which may lead to repeated episodes of the disease and continuous deterioration of lung function. The development of the disease is unaffected by conventional bronchodilators and ultimately results in a surge in mortality [6, 7]. Due to the high cost of treatment of COPD and the rising number of hospitalized COPD patients in the past decade, the academic community has deepened research on the treatment of stable COPD, aiming to reduce the number of acute attacks, improve patients' exercise capacity, and lessen their medical burden [8, 9]. Regardless of the stage of COPD, patients can benefit from exercise training to improve their exercise tolerance and boost pulmonary rehabilitation. Traditional Chinese medicine (TCM) exercises are the main method of TCM pulmonary rehabilitation, with advantages of low costs, safety, and less restriction, which can gradually restore the function of the internal organs, alleviate the inflammatory response, and mitigate the clinical symptoms during the training process. Liuzijue breathing exercise is a widely used exercise therapy in clinical practice that has been proven to be rewarding in the enhancement of a patient's respiratory function [10, 11]. However, it also requires adjuvant treatment methods to aggrandize its curative effect owing to its varying application effect. Acupoint catgut embedding therapy is a new type of discipline that combines thousands of years of experience in acupuncture in traditional Chinese medicine and over 30 years of experience in embedding therapy, which can dredge the meridians by generating physiological, physical, and chemical stimuli to acupoints to cure chronic diseases. Clinical research has shown that acupoint catgut embedding therapy yields quick-acting, long-acting, and targeted effects for certain chronic and miscellaneous diseases. Nonetheless, few scholars have been able to draw on any systematic research into the combined use of acupoint catgut embedding therapy with Liuzijue breathing exercise, which requires more clinical evidence for its efficacy and standardization.

Accordingly, this study applied Liuzijue breathing exercise combined with acupoint catgut embedding therapy for patients with stable COPD, aiming to analyze and explore the effect of Liuzijue breathing exercise combined with acupoint catgut embedding therapy on the pulmonary rehabilitation of patients with stable COPD. The report is as follows.

## 2. Materials and Methods

### 2.1. General Information

For the calculation of the sample size, *G*^*∗*^Power 3.1.9.7 was used, and a priori power analysis was calculated to achieve a statistical power of 95% with a level of significance *α* = 5%, the sample size of each group is 34. A total of 100 patients with COPD admitted to our hospital from February 2020 to February 2021 were included and assigned to the experimental group (*n* = 50) and the control group (*n* = 50) according to the order of admission. The two groups did not differ regarding the general information (*P* > 0.05). This study was approved by the ethics committee of the hospital.

### 2.2. Inclusion Criteria

The inclusion criteria include the following: (1) the patients and their families signed the informed consent form after being fully informed of the purpose and process of the study; (2) the patients' symptoms, signs, laboratory, and imaging examinations met the relevant diagnostic criteria in the “Guidelines for the Diagnosis and Treatment of Chronic Obstructive Pulmonary Disease (2013 Revised Edition) (1)”; (3) TCM diagnosis met the relevant diagnostic criteria in the “Guidelines for the Diagnosis and Treatment of Chronic Obstructive Pulmonary Disease (2011 Edition),” and patients were categorized into the spleen-lung-qi deficiency type or the lung-kidney-qi deficiency type [12]; (4) patients with a stable stage of disease confirmed by imaging examination, and no acute episode of COPD occurred in the past 4 weeks [13]; (5) those in stage II and III COPD; (6) those aged less than 80 years; (7) patients with complete clinical data could fully participate in the treatment.

### 2.3. Exclusion Criteria

Patients were excluded if they had one of the followings: (1) a history of heart failure within 3 months before the enrollment; (2) acute and chronic pneumonia, tuberculosis, pulmonary edema, and other lung diseases; (3) serious diseases in the liver, kidney, cardiovascular and cerebrovascular, and blood system; (4) pregnancy or breastfeeding; (5) with cognitive or communication disorders or mental illness; (6) poor treatment compliance or incomplete clinical data; (7) currently participating in other research.

### 2.4. Methods

Control group: patients received convention treatment and were given inhalation of budesonide and formoterol fumarate powder for inhalation (AstraZenecaAB, NMPA approval number: H20140457, 320 ug/bottle), twice a day, one inhalation per time. Daily oxygen therapy was performed for 15 hours, and oxygen was continuously provided during night sleep. The patient was treated continuously for 4 weeks.

Experimental group: based on the control group, Liuzijue breathing exercise combined with acupoint catgut embedding therapy was provided. Liuzijue breathing exercise: under the guidance of a specific nurse, the patients were instructed to stand with feet shoulder-width apart, waist and hips relaxed, and knees slightly bent, perform diaphragmatic breathing with inhalation through the nose and exhalation through the mouth, produce the six sounds (xū, hē, hū, sī, chuī, and xī) during exhalation, and move the weight to the heel while doing the sphincter exercise. After the six sounds were repeated six times, the patients rested till normal breathing, followed by another round of exercise, twice a day, once in the morning and once in the evening, 30 minutes per time, continuous treatment for 4 weeks.

Acupoint catgut embedding therapy: the therapy was performed by an acupuncturist. After the skin sterilization, local anesthesia was performed at an area of 1 cm–2 cm of the acupoints (BL13, BL20, and BL23) to be embedding catgut. A skin suture needle with absorbable surgical sutures, with the skin between the two local anesthetic points pinched, was pierced from one side of the local anesthetic point, through the muscle layer or subcutaneous tissue, to the opposite local anesthetic point. The catgut was then cut at both ends with the catgut completely under the skin after the skin was relaxed. Sterile dry cotton balls (swabs) were applied to stop bleeding, with a sterile dressing to protect the wound for 3 d–5 d. The embedding therapy was performed once every two weeks, with continuous treatment for 4 weeks.

### 2.5. Observational Indexes

The patient's parameter indicators were tested before the start of the experiment and at the end of the experiment (4 weeks).

1. Clinical efficacy: clinical symptoms including sputum expectoration, coughing, wheezing, shortness of breath, and spontaneous sweating were divided into normal (0 points), mild (1 point), moderate (2 points), and severe (3 points) according to the severity. Basically cured: the clinical symptoms have basically disappeared, and the symptom score reduction rate reached 95% and above; significantly effective: the clinical symptoms were improved significantly, and the symptom score reduction rate was 70% to 94%; effective: the clinical symptoms were partially improved, and the symptom score was reduced by 30% to 69%; ineffective: the clinical symptoms were not improved or even worsened, and the symptom score was reduced below 30%.
2. Lung function indicators: a pulmonary function meter (Beijing Maibang Photoelectric Instrument Co., Ltd., Beijing Drug Administration and Machinery Zhunzi 2009 no. 2210235) was used to measure the patient's forced vital capacity (FVC), forced expiratory volume in one second (FEV1), and FEV1/FVC.
3. Activities of daily living (ADL) scores and quality of life (QOL) scores: the ADL score includes eating, bathing, grooming, dressing, stool control, urination control, toilet use, bed and chair transfer, walking, and stairs. Each item is scored between 0–10 points, and the total score is between 0–100 points. A score of 60 or more: basic self-care, 41–60: assistance in daily life, 20–40: great assistance in daily life, and a score below 20: full-time care is required [14]. The QOL score includes appetite, spirit, and sleep. The total score is between 0 and 60 points, and the lower the score, the worse the quality of life of the patient [15].
4. TCM syndrome points: the main symptoms include cough, sputum expectoration, wheezing, and shortness of breath. The score ranges from 0 to 4 points, which can be divided into normal (0 points), mild (1 point), moderate (2 points), and severe (3 points) [16].
5. number of acute exacerbations: the patient had 1 major symptom (dyspnea, increased sputum volume, and purulent sputum), accompanied by a major or minor symptom (cold, sore throat, and cough), with a duration of over 2 days.
6. Medical expenses: it includes medicine fee, inspection fee, laboratory examination fee, material fee, and treatment costs.
7. Adverse reactions: it includes skin rash, skeletal muscle tremor, headache, throat discomfort, and sinus tachycardia.
8. Immune indicators: 5 ml of the patient's morning venous fasting blood was collected and processed by flow cytometry (Aisen Bio-Hangzhou Co., Ltd., Zhejiang Food and Drug Administration Approval Number 2014: 2400581) to detect the patient's *T* lymphocyte subsets level (CD3+, CD4+, and CD8+).

### 2.6. Withdrawal Criteria

The withdrawal criteria include the following: (1) those with adverse events or serious adverse events; those who were not suitable to continue the experiment; (2) those whose condition deteriorated during the experiment were considered not suitable for further experiment according to the doctor's evaluation; (3) those with serious comorbidities or complications, and special physiological changes, which were not suitable for the experiment according to the doctor's evaluation; (4) the patients were unwilling to continue the clinical trial and asked for withdrawal.

Regardless of the above reasons, for the cases who withdrew from the trial, their records were retained, with the last test results as the final results, and the efficacy and adverse reactions analysis were also conducted to obtain relevant data.

### 2.7. Statistical Processing

The data obtained in this study were processed by software SPSS 20.0, and the graphics plotting was processed using GraphPad Prism 7 (GraphPad Software, San Diego, USA) software. Continuous variables are presented as the mean. Baseline characteristics were compared between the two groups by an unpaired *t*-test for continuous variables and by chi-squared analysis or Fisher's exact test. A two-way repeated measures analysis of variance (ANOVA) was used to analyze the differences between before and after treatment. *P* < 0.05 indicates statistical significance.

## 3. Results

### 3.1. Comparison of Clinical Efficacy

Between February 2020 and February 2021, a total of 100 patients with stable COPD at our hospital were enrolled in this study and were randomized to the experimental group (*n* = 50) and control group (*n* = 50). All enrolled patients were included for further analysis. The baseline characteristics of the subjects are shown in Table 1. There were no significant differences between the two groups in any patient characteristics such as gender, age, weight, BMI, and arterial blood gas index.

**Figure 1:**
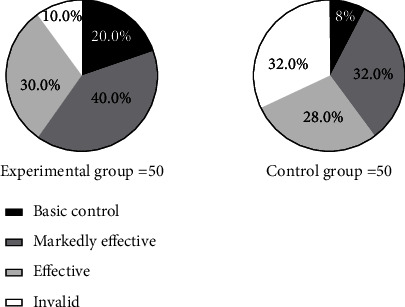
Comparison of clinical efficacy of patients [*n* (%)]. Note: the left pie chart in Figure 1 is the experimental group, and the right pie chart is the control group. The black area in the figure is basically cured, the dark gray area is significantly effective, the light gray area is effective, and the white area is ineffective. The number of basically cured, significantly effective, effective, and ineffective patients in the experimental group were 10, 20, 15, and 5, respectively; the number of basically cured, significantly effective, effective, and ineffective patients in the control group were 4, 16, 14, and 16, respectively.

The pie chart was used to compare the clinical efficacy of the two groups of patients. The proportion of basically cured, significantly effective, effective, and ineffective patients in the experimental group was 20%, 40%, 30%, and 10% respectively, and the overall effective rate was 90%. The proportion of basically cured, significantly effective, effective, and ineffective patients in the control group was 8%, 32%, 28%, and 32% respectively, and the overall effective rate was 68%. It can be concluded that the effective rate of treatment in the experimental group (90%) was significantly higher than that in the control group (68%) (Figure 1).

### 3.2. Comparison of Lung Function Indicators

Before treatment, there was no significant difference in lung function indicators between the experimental group and the control group (*P* > 0.05). Statistical analysis of lung function indicators between the two groups after treatment (*T*-test) showed that FVC, FEV1, and FEV1/FVC were statistically different (*P* < 0.05), and pulmonary function improvement in the experimental group was better than that in the control group (Table 2).

### 3.3. Comparison of ADL and QOL Scores

**Figure 2:**
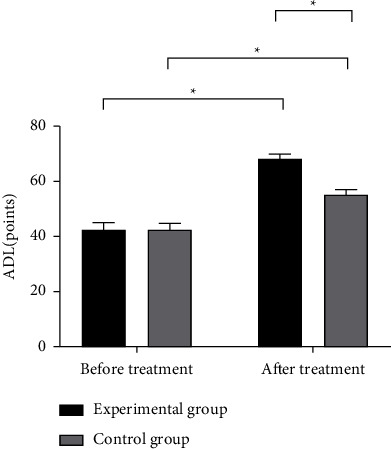
Comparison of ADL scores of patients ($\bar{x}\pm s$, points). Note: the abscissa in Figure 2 is before and after treatment, and the ordinate is ADL (points) from left to right; the black area in the figure is the experimental group, and the gray area is the control group; ^*∗*^means *P* < 0.001. The ADL scores of the experimental group and the control group before the treatment were not significantly different (42.52 ± 2.45 vs. 42.39 ± 2.38, *P* > 0.05); the ADL scores of the experimental group after the treatment were significantly higher than those of the control group (68.31 ± 1.54 vs. 55.17 ± 1.96, *P* < 0.001).

**Figure 3:**
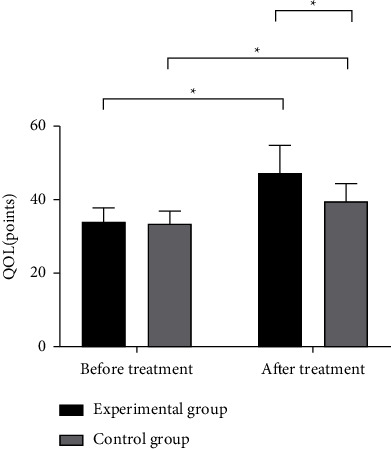
Comparison of QOL scores of patients ($\bar{x}\pm s$, points). Note: in Figure 3, the abscissa is from left to right before and after treatment, and the ordinate is QOL (points); the black area in the figure is the experimental group, and the gray area is the control group; ^*∗*^means *P* < 0.001. The QOL scores of the experimental group and the control group before the treatment were not significantly different (34.14 ± 3.63 vs. 33.37 ± 3.56, *P* > 0.05); the QOL score of the experimental group after the treatment was significantly higher than that of the control group (47.42 ± 7.46 vs. 39.59 ± 4.87, *P* < 0.001).

The ADL and QOL scores of the two groups were statistically analyzed before and after treatment, and the results showed that there was no statistical difference in the ADL and QOL scores of the two groups before treatment (*P* > 0.05), after treatment, ADL, and QOL scores in the experimental group were significantly higher than those in the control group, and the differences were statistically significant (*P* < 0.05) (Figures 2 and 3).

### 3.4. Comparison of TCM Syndrome Scores

**Figure 4:**
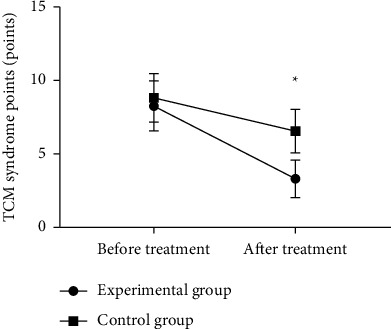
Comparison of TCM syndrome scores of patients ($\bar{x}\pm s$, points). Note: n Figure 4, the abscissa is from left to right before and after treatment, and the ordinate is the TCM syndrome score (points); the dotted line in the figure is the experimental group, and the square line is the control group; ^*∗*^means *P* < 0.001. There was no significant difference in the TCM syndrome scores of the experimental group and the control group before the treatment (8.28 ± 1.71 vs. 8.86 ± 1.65, *P* > 0.05); the TCM syndrome scores of the experimental group after the treatment were significantly lower than those of the control group (3.32 ± 1.28 vs. 6.57 ± 1.48, *P* < 0.001).

The results showed that TCM syndrome scores of the experimental group were significantly lower than that of the control group (*P* < 0.05), the efficacy of the two groups was evaluated from the perspective of traditional Chinese medicine, and the clinical symptoms of the patients were significantly improved after treatment (Figure 4).

### 3.5. Comparison of Acute Exacerbations

**Figure 5:**
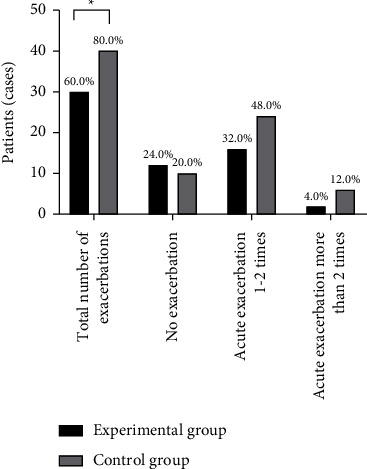
Comparison of the number of acute exacerbations in patients [*n* (%)]. Note: in Figure 5, the abscissa from left to right is the total number of acute exacerbations, the number of patients without acute exacerbations, the number of patients with 1–2 acute exacerbations, and the number of patients with more than 2 acute exacerbations; the ordinate is patients (cases); the black area in the figure is the experimental group, and the gray area is the control group; ^*∗*^means *P* < 0.05. The number of patients with acute exacerbation in the experimental group was significantly lower than that in the control group (30 vs. 40, *P* < 0.05); the number of patients without acute exacerbation, the number of patients with acute exacerbation 1–2 times, and the number of patients with acute exacerbation more than 2 times in the experimental group were not significantly different from those in the control group (12 vs. 10, 16 vs. 24, 2 vs. 6, *P* > 0.05).

Statistical analysis was conducted on whether there were acute exacerbations in the experimental group and the control group. The results showed that the number of acute exacerbations in the experimental group (0.1–2>2) was lower than that in the control group, indicating that the experimental method in this paper can significantly reduce the number of acute exacerbations in the hospital and maintain clinical stability (Figure 5).

### 3.6. Comparison of Medical Expenses

The results showed that the treatment cost of the experimental group, including medicine fee, inspection fee, laboratory examination fee, material fee, and treatment cost, was lower than that of the control group (*P* < 0.05). The overall cost of the experimental group was lower, which could significantly reduce the medical burden on patients (Table 3).

### 3.7. Comparison of the Incidence of Adverse Reactions

The chi-square test was used to compare the incidence of adverse reactions between the experimental group and the control group, and the results indicated that no serious adverse reactions occurred between the two groups, and there was no statistical difference in the incidence of adverse reactions (*P* > 0.05) (Table 4).

### 3.8. Comparison of Immune Indicators

*T* lymphocyte subsets of the experimental group and the control group were statistically analyzed. In the experimental group, the amount of CD3^+^ was significantly increased (*P* < 0.001) and also increased in the control group (*P* < 0.05). The amount of CD3^+^ in the experimental group was significantly higher than that in the control group (*P* < 0.001). In the experimental group, the amount of CD4^+^ was significantly increased (*P* < 0.001) but wasn't increased in the control group (*P*=0.088). The amount of CD4^+^ in the experimental group was significantly higher than that in the control group (*P* < 0.001). In the experimental group, the amount of CD8^+^ was significantly increased (*P* < 0.001) but wasn't increased in the control group (*P* < 0.001). The amount of CD8^+^ in the experimental group was significantly higher than that in the control group (*P* < 0.001) (Table 5).

In conclusion, compared with the control group, the experimental group can improve the clinical efficacy, improve the lung function and clinical symptoms of patients, improve the quality of life, reduce the number of acute exacerbations, appropriately reduce the treatment cost and improve immune function, but there is no statistical difference in the incidence of adverse reactions between the two groups.

## 4. Discussion

With the continuous upgrading of medical technology, the incidence of COPD in China has shown a steady downward trend in the past decade, while its hospitalization rate has been witnessing an increase. Research has shown that the treatment cost of a single COPD patient accounts for about 48% of the total family income, which is an extremely heavy medical burden [17]. As of 2017, the death cases of COPD in China have reached more than one million every year [18], with its mortality rate ranking first in the world [19], indicating a high risk of death accompanied by enormous medical expenses and a gloomy overall survival. The reason for the high mortality of patients lies in their neglect of COPD treatment at the stable stage, after which the disease may experience repeat onset and result in apparently impaired lung function, with frequent occurrence of various complications and critically undermined treatment effect. Therefore, the enhancement of treatment during the stable period of COPD is the key to the improvement of the patient's survival.

Currently, the treatment of stable COPD in China is mostly single-drug therapy, with bronchodilators as the treatment drugs recommended by COPD treatment guidelines at home and abroad, including glucocorticoids, *β*2 adrenergic receptors agonists, anticholinergics, and theophylline drugs. However, no effective drugs have been discovered to improve the survival of patients; moreover, some drugs may fail to ameliorate the lung function of patients with limited effect. Hence, the transition from single drug control to drug control combined with pulmonary rehabilitation training is imperative for stable treatment. Regardless of the patient's disease stage, exercise tolerance can be improved through exercise training to improve the quality of life and achieve pulmonary rehabilitation. The Liuzijue breathing exercise selected in this study was proposed by Tao Hongjing in the Southern and Northern Dynasties. It is performed by producing 6 different sounds, (“xū,” “hē,” “hū,” “sī,” “chuī,” and “xī”) through expiration together with corresponding body movements to realize the effects of exercising viscera, regulating qi and blood, balancing yin and yang, nourishing lung qi, dispelling stagnation, and strengthening health, to combine breathing with thoughts and simultaneous movement of breathing muscles and limbs, to achieve the coordination of multiple organs in treatment. Previous research has shown that Liuzijue breathing exercise can alleviate the clinical symptoms of patients [20]. This study also reached the same conclusion. The experimental group obtained superior lung function indexes and TCM syndrome scores to those of the control group (*P* < 0.05), and the patient's immune function was remarkably improved, indicating that this treatment method optimizes the patient's organ function and relieves their cough and asthma. However, a prior study has also demonstrated the varying effects of Liuzijue breathing exercises on different patients [21]; the enhancement of the clinical efficacy of Liuzijue breathing exercises remains one of the most pressing clinical issues to be addressed.

Based on the Liuzijue breathing exercise, this study adopted acupoint catgut embedding therapy as an auxiliary treatment measure which also has a wide range of applications. After embedding, the catgut will gradually soften, decompose, and liquefy to be absorbed by the body, which produces physiological, physical, and chemical stimulation to the acupoint for up to 20 days, thereby exerting a slow, soft, long-lasting, and benign “long-acting acupuncture effect,” to dredge the meridians and achieve the effect of curing chronic diseases. In this study, acupoint catgut embedding was performed on the bilateral BL13, BL20, and BL23 of the experimental group. Results showed a significantly higher treatment effective rate and a remarkably lower number of acute exacerbations, which indicates the promising performance of the combination of the two therapies.

Scholar Siqin et al. performed Liuzijue breathing exercises and acupoint massage for patients with stable COPD and confirmed that the ADL score of the experimental group after treatment was (69.68 ± 2.31) points, and the QOL score was (50.11 ± 2.10) points, both of which were significantly better than those of the control group (*P* < 0.05) [22, 23], suggesting the advantages of long-term and specific effects of acupoint therapy on COPD. Moreover, the body's response to traditional Chinese medicine therapy is rather mild, which has been proven by the absence of serious adverse reactions during the treatment. Same results were also obtained in this study. It is worth noting that the study by Siqin et al. showed that the medical expenses of the experimental group with additional TCM therapy were slightly lower than those of the control group without significant difference. Results of this study demonstrated that the medical expenses of the experimental group are significantly lower than the control group, which is speculated to be related to the differences in regions and specific treatment protocols. Whether the comprehensive treatment can fully reduce the medical burden of COPD patients in China requires further research. The article did not discuss the effects of the season on COPD, it is the study's limitations. Larger sample sizes are needed in the future to prove the findings.

In conclusion, acupoint catgut embedding therapy combined with Liuzijue breathing exercise, with high safety, can improve the treatment effect and the quality of life of patients with stable COPD, which merits clinical promotion.

## Figures and Tables

**Table 1:** Comparison of general information of patients.

Groups	The experimental group (*n* = 50)	The control group (*n* = 50)	*X* ^2^/*t*	*P*
Gender			0.060	0.806
Male	39	40		
Female	11	10		

Age (y)				
Range	52–76	54–74		
Average age	68.21 ± 2.65	68.26 ± 2.54	0.096	0.924
Average weight (kg)	55.21 ± 2.14	55.23 ± 2.65	0.042	0.967
BMI (kg/m^2^)	21.45 ± 2.15	21.50 ± 2.23	0.114	0.909

Treatment department				
Respiratory department	5	6	0.102	0.749
Emergency department	44	42	0.332	0.564
Others	1	2	0.344	0.558

Operation			0.154	0.695
Yes	4	3		
No	46	47		

Arterial blood gas index (mmHg)				
PaO_2_	55.21 ± 6.98	55.34 ± 6.54	0.096	0.924
PaCO_2_	56.12 ± 6.41	56.23 ± 6.25	0.087	0.931

Place of residence			0.040	0.841
Urban	28	27		
Rural	22	23		

Monthly income (yuan)			0.040	0.841
≥4000	25	24		
<4000	25	26		

Living habit				
Smoking	45	46	0.122	0.727
Drinking	34	35	0.047	0.829

Education level			0.040	0.841
High school and below	26	27		
Junior college and above	24	23		

**Table 2:** Comparison of lung function indicators of patients ($\bar{x}\pm s$).

Indicators	The experimental group		The control group		*t*	*P*
FVC (L)	Before treatment	2.09 ± 0.61	Before treatment	2.13 ± 0.63	0.323	0.748
	After treatment	3.23 ± 0.98	After treatment	2.68 ± 0.82	3.044	<0.05
	*t*	6.983	*t*	3.761		
	*P*	<0.001	*P*	<0.05		

FEV_1_ (L)	Before treatment	1.69 ± 0.46	Before treatment	1.75 ± 0.54	0.598	0.551
	After treatment	2.42 ± 0.85	After treatment	2.03 ± 0.76	2.419	<0.05
	*t*	5.341	*t*	2.124		
	*P*	<0.001	*P*	<0.05		

FEV_1_/FVC (%)	Before treatment	67.12 ± 2.43	Before treatment	66.83 ± 2.38	0.603	0.548
	After treatment	74.18 ± 2.85	After treatment	70.51 ± 2.66	6.657	<0.001
	*t*	12.329	*t*	7.290		
	*P*	<0.001	*P*	<0.001		

**Table 3:** Comparison of patient's medical expenses ($\bar{x}\pm s$, yuan).

Groups	Medicine fees	Inspection fees	Lab fees	Material fees	Treatment costs
The experimental group	4457.32 ± 235.68	501.65 ± 45.68	1210.68 ± 125.32	542.65 ± 52.63	1426.98 ± 210.32
The control group	4612.65 ± 236.98	657.12 ± 50.12	1359.65 ± 120.57	657.98 ± 45.95	1566.98 ± 235.65
*t*	3.286	16.211	6.057	11.672	3.134
*P*	0.001	<0.001	<0.001	<0.001	0.002

**Table 4:** Comparison of the incidence of adverse reactions in patients [*n* (%)].

Groups	Rash	Skeletal muscle fibrillation	Headache	Throat discomfort	Sinus tachycardia
The experimental group	3 (6.0)	2 (4.0)	3 (6.0)	2 (4.0)	0 (0.0)
The control group	4 (8.0)	3 (6.0)	4 (8.0)	3 (6.0)	1 (2.0)
*X* ^2^	0.154	0.211	0.154	0.211	1.010
*P*	0.695	0.646	0.695	0.646	0.315

**Table 5:** Comparison of immune indicators of patients ($\bar{x}\pm s$, %).

Indicators	The experimental group		The control group		*t*	*P*
CD3^+^	Before treatment	49.21 ± 5.23	Before treatment	49.25 ± 5.21	0.038	0.970
	After treatment	59.26 ± 4.21	After treatment	52.56 ± 4.10	8.062	<0.001
	*t*	10.585	*t*	3.530		
	*P*	<0.001	*P*	<0.05		

CD4^+^	Before treatment	35.68 ± 2.65	Before treatment	35.26 ± 2.45	0.823	0.413
	After treatment	39.65 ± 2.44	After treatment	36.12 ± 2.54	27.163	<0.001
	*t*	7.793	*t*	1.723		
	*P*	<0.001	*P*	0.088		

CD8^+^	Before treatment	28.65 ± 2.10	Before treatment	28.65 ± 2.13	0.000	1.000
	After treatment	24.15 ± 1.22	After treatment	25.68 ± 2.08	4.487	<0.001
	*t*	13.102	*t*	7.054		
	*P*	<0.001	*P*	<0.001		

## Data Availability

The datasets used and analyzed by this study are available from the corresponding author on reasonable request.
